# Maternal experiences of caring for preterm infants in a vulnerable South African population

**DOI:** 10.4102/hsag.v26i0.1549

**Published:** 2021-07-30

**Authors:** Kristen Buys, Berna Gerber

**Affiliations:** 1 Department of Speech, Language and Hearing Therapy, Faculty of Medicine and Health Sciences, Stellenbosch University, Cape Town, South Africa

**Keywords:** preterm birth, mother, early intervention, speech-language therapy, low socio-economic, feeding methods, culture, social support

## Abstract

**Background:**

Caring for a preterm infant is known to be a stressful experience as these infants are at a high risk of medical sequelae and developmental delays. Early intervention is imperative for the best developmental outcome for the infant. Such interventions are often delivered through the mother or primary caregiver; however, healthcare professionals are seldom aware of all the factors that influence maternal well-being, potentially influencing her ability to provide optimal care.

**Aim:**

To explore the experiences of a group of vulnerable women, namely, isiXhosa-speaking mothers of preterm infants living in low socio-economic circumstances in the Western Cape province of South Africa, regarding having, caring for and feeding their preterm infants within the first 6 months of the infant’s life.

**Setting:**

A follow-up clinic for preterm infants and their mothers at a public tertiary hospital in Cape Town, South Africa.

**Methods:**

The study employed a qualitative, cross-sectional design that was explorative and contextual in nature. A discussion schedule was used to guide 15 in-depth interviews with mothers that were later thematically analysed.

**Results:**

Social support and religion positively influenced maternal coping. The infant’s medical stability was the main concern for mothers and concerns regarding the infant’s development did not arise. Prematurity influenced mothers’ decisions to use traditional medicines and hospital care affected some traditional practices.

**Conclusion:**

The study findings highlighted the influence of traditional and religious beliefs, the importance of the cultural education of medical staff members and a support system to improve maternal experiences.

**Contribution:**

The findings provide insights into maternal experiences with implications for healthcare practitioners’ continued education in an ethnically diverse setting.

## Introduction

Preterm births account for a growing 5% – 18% of all births worldwide, with more preterm infants surviving at younger gestational ages (WHO 2018 [Fact Sheet]). It is well established that preterm infants are at increased risk – two to three times that of their full-term counterparts – of developmental difficulties and medical sequelae (Soleimani, Zaheri & Abdi 2014). Literature suggests that such difficulties range across health and neurodevelopmental domains. Neurodevelopmental difficulties may affect many areas of development, such as feeding, cognitive and social development, including attachment and communication (Hee Chung, Chou & Brown 2020; Johnson et al. 2015). Furthermore, research studies have linked higher incidences of preterm birth with lower socio-economic circumstances, as 60% of preterm births occur within the Global South (Blencowe et al. 2012; Boutayeb et al. 2019; WHO 2018). In South Africa, approximately half of the population live in low socio-economic circumstances (Statistics South Africa 2020). The low socio-economic status (SES) population experiences specific risk factors pertaining to their health, such as increased exposure to human immunodeficiency virus and acquired immunodeficiency syndrome (HIV and AIDS) and tuberculosis, poor housing conditions and poor sanitation (Arroyave et al. 2014). Such external factors may negatively influence a vulnerable preterm infant’s health and development, not only directly but also through risk to the well-being of the mother or primary caregiver who may not be able to provide optimal care (Black et al. 2017; Van Schalkwyk et al. 2020). The aforementioned risk factors that may affect the development of a preterm infant are likely to be present as the infant matures into a toddler and later into a child of school-going age.

With many factors potentially negatively influencing an infant’s development in low SES households, early childhood intervention (ECI) is crucial for mitigating developmental delays and the associated effects on future aspects of the child’s life, such as academic and social functioning (Richter et al. 2019; Smythe et al. 2020). Early childhood intervention efforts with infants are best targeted at the level of the mother or primary caregiver of the infant. The infant’s mother is instrumental in the engagement of protective factors and mediation of risk factors, such as providing language stimulation to prevent or mediate a communication developmental delay (Bayrakli & Sukuoglu 2019; Ingber et al. 2010). Additionally, it is well known that positive mother–infant interactions foster optimal developmental environments (Festante et al. 2019).

**FIGURE 1:**
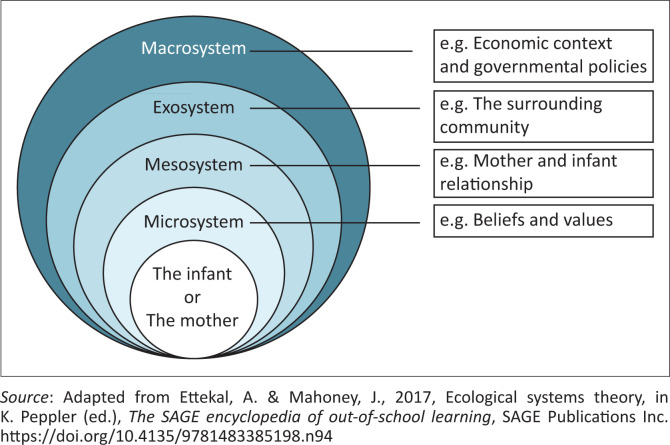
Bronfenbrenner’s bio-ecological model of child development.

Factors influencing childhood development can be seen as either ‘risk’ or ‘protective’ in nature, as illustrated by Pascoe, Bissessur and Mayers’s (2016) application of the transactional model on childhood development. Furthermore, risk or protective influences on infant development and well-being can be viewed as existing within different levels of influence, such as those systems described by Bronfenbrenner’s (1989) bio-ecological model (see Figure 1). Bronfenbrenner’s bio-ecological model is based on the understanding that the relationship between an infant and a caregiver influences the infant’s development and vice versa. Furthermore, infant development and the parent–infant relationship are affected by external influences, such as the parents’ employment, the surrounding community and broader social and political factors. These influences are organised into various ‘systems’ (see Figure 1). Nevertheless, influences affecting an infant at such a young age are mainly experienced through the mother or primary caregiver.

Caring for a preterm infant is often a stressful experience, which places mothers at a higher risk for psychiatric conditions such as anxiety and depression (Steyn, Poggenpoel & Myburgh 2017). Maternal psychiatric conditions may persist long after giving birth, and mothers with high stress may experience difficulty in the mother–infant relationship (Gonçalves et al. 2020). Difficulties with mother–infant interactions or attachment bonds may affect the long-term socio-emotional development of the infant, lead to higher incidence of maternal neglect and are associated with poorer child outcomes (Choi et al. 2017; Lehnig et al. 2019). Maternal well-being is, therefore, an essential part of ECI efforts, but one that is often neglected. The following section will discuss ECI within the South African context.

Although there is increased recognition of the importance of ECI, there still exist multisectoral constraints within the implementation and improvement of such programmes (Pelletier & Neuman 2014). One important consideration in designing effective and culturally congruent childhood interventions is that of maternal beliefs and values. If interventions are incongruent with maternal values and practices, the aims and methods are not likely to carry over to the home environment, possibly leading to ineffective interventions (Jones et al. 2017; Krummer, Lopez-Reyna & Hughes 2007). In recognition of this need for culturally congruent interventions, the Professional Board for Speech, Language and Hearing Professions of the Health Professions Council of South Africa (HPCSA) developed a guideline outlining the speech-language pathologist’s (SLP’s) duty to ‘…ensure that their practice is consistently responsive to the cultural and linguistic backgrounds of their clients…’ (HPCSA 2019:6–7). However, SLPs, as well as other healthcare professionals in the South African context, are often not equipped with the cultural and linguistic knowledge necessary to fulfil this duty (Southwood & Van Dulm 2015). A lack of information about local people, cultures and language contributes to possible incongruences between healthcare professionals and the patients whom they serve (Pascoe et al. 2017). Thus, there appears to be knowledge gaps pertaining to the local people, cultures and language within South Africa, with a specific reference to the experiences shared by mothers of infants living in low socio-economic circumstances. In order for healthcare professionals to provide culturally congruent intervention services, access to information about the local experiences is essential in building an understanding of clients’ beliefs, needs and circumstances.

The current study focussed on investigating maternal experiences of a vulnerable group of women who are not well represented in the current research literature, those belonging to the second-largest ethnic group in South Africa, namely, isiXhoxa-speaking mothers. The aim of the study was to describe the above-described mothers’ experiences with regards to having, caring for and feeding their preterm infants (birth to 6 months of age) within low socio-economic circumstances. The study adopted a theoretical approach that reflected a combination of Bronfenbrenner’s bio-ecological systems model of childhood development (Figure 1; Bronfenbrenner 1989) as well as the transactional model (Sameroff 2009) applied to childhood development (Pascoe et al. 2016). Thus, this study explored ‘protective’ and ‘risk’ factors acting at different systems of influence that may affect childhood well-being and development through the experience of the mother.

## Methodology

### Study design

This study employed a qualitative, cross-sectional design and was explorative, descriptive and contextual in nature. The phenomenological tradition was followed (Creswell 2007). The study used in-depth individual interviews guided by a semi-structured discussion schedule consisting mainly of open-ended questions.

### Setting

The interviews between the author and the isiXhosa-speaking mothers, from the surrounding urban low socio-economic communities, were conducted in a private office at a paediatrics follow-up clinic within a tertiary hospital in the Western Cape province.

### Participants and sampling strategy

A purposive maximum variation sampling was used to recruit participants who met the inclusion criteria. The sampling strategy made it possible to identify and include participants who have experienced the same phenomenon, but whose background and biological information is slightly different, so that a range of the specified population could be represented in the study’s results. The data were monitored and analysed throughout the collection process and data saturation was reached after 15 interviews (as determined by the measurements described by Malterud et al. 2016).

Participants were recruited by the first author if they were 18 years or older and they were isiXhosa first language mothers (and the primary caregivers) of infants who met the following criteria:

- Premature, as defined by the WHO (2018 [Fact Sheet]) as born alive before 37 completed gestational weeks and with a birth weight below 2500 g.
- Three to six months chronological age (thus 0–3 months corrected age), this was to allow time for the participants to experience early infancy at the hospital and at home.
- Medically stable without any known biological, physical, congenital or neurological disorders at the time of the interview. For example, infants with known medical complications, such as suffering a cerebrovascular accident, a heart defect or bronchopulmonary dysplasia, were excluded as these conditions may be accompanied by specific difficulties in caregiving. Because the aim of the study was specifically to explore the experience of caring for a preterm infant, and not an infant with a diagnosis, mothers of infants with known medical complications were excluded.

Participants were to be of ‘low-socioeconomic status’, as determined by the educational attainment of no more than an NQF level 4, and a combined household income of less than R100 000. Household income is determined and classified upon hospital admission and documented in each patient’s medical file.

A Health Research Ethics Committee as well as the Western Cape Health Research Committee gave permission to recruit potential participants with the assistance of the paediatrician in charge of the clinic. The paediatrician consulted her own appointment book to identify days where potential participants would be attending the clinic. No formal or informal relationship exists between this paediatrician and the researchers, apart from their shared involvement at a tertiary hospital that acts as a training site for the university. Potential participants were identified at the research site and were then invited to participate in the study by the first author. Fifteen interviews constituted the final data set. Participants came from various surrounding communities. Six participants lived with their partner and father of the preterm infant, and in some cases, their other children. Nine of the participants resided with family, including parents, cousins and siblings. A description of the participants is presented in Table 1.

**TABLE 1:** Participant descriptions.

Participant number	Age of participant	Ages of participants’ other children (years)	Preterm infants’ chronological age (months)	Preterm infants’ corrected age	Preterm infants’ age at birth (weeks)	Household composition	Participants’ employment status	Participants’ marital status
1	22	NA	4	3 weeks	26	Participants’ mother and sister	Unemployed	Single
2	34	NA	3.5	1.5 months	29	Participants’ sister	Employed	Single
3	26	9	5	3 months	28–29	Partner	Employed	Single
4	24	NA	5	3 months	33	Participants’ parents and five other family members	Unemployed	Single
5	26	10	5	3 months	31	Partner	Unemployed	Single
6	29	NA	2	Term	29	Participants’ brother	Unemployed	Single
7	35	NA	6	2 months	22*	Participants’ brother and sister in-law and their toddler	Unemployed	Single
8	36	4	2	Term	28	Participants’ sister, brother in-law and their child	Unemployed	Single
9	24	NA	3	Term	27–28	Participants’ three brothers and cousin	Employed	Single
10	36	14; 10	3	2 weeks	29	Participants’ two older children and niece	Unemployed	Single
11	26	NA	5.5	3 months	28–31	Participants’ parents, brother, sister and her sisters’ three children	Unemployed	Single
12	24	NA	5	3 months	32	Participants’ parents, four older family members and her niece	Unemployed	Single
13	26	5	6	3 months	27	Partner and her other child	Unemployed	Single
14	35	14; 9	4	2 months	32	Partner and their older child	Employed	Married
15	30	7; 4	3	1 month	31	Participants’ father and her two other children	Unemployed	Single

NA, Not applicable.

* , gestational age of preterm infant at birth as estimated by the mother, which could not be confirmed by medical file.

### Data collection

The first author collected the data. Potential participants arrived for their infant’s medical follow-up appointment at the hospital where the infant was born. Upon conclusion of the medical consultation, the author was introduced to the potential participant by the consulting paediatrician. The author then conversed privately with the candidate in English to establish rapport, informed the candidate about the study and invited her to participate. In the cases of a language barrier, a trained interpreter conversed with the potential participant in isiXhosa. The potential participant was then given an opportunity to ask questions about the research and to consider participation. If the candidate accepted the invitation, the author took her to a private consultation room where the participants were read the information and consent form in either English by the author or isiXhosa by the interpreter, and given the opportunity to ask further questions about the study or for clarification. The candidate was then given another opportunity to indicate whether she wanted to participate or not. If verbal consent to participate was obtained, this was followed by obtaining written consent and the interview commenced. A total of 15 interviews were conducted over a period of 4 weeks.

Each interview was semi-structured in nature and guided by a discussion schedule compiled by the authors after careful review of relevant literature and questionnaires used in similar studies. The discussion schedule consisted largely of open-ended questions related to the aims of the study. The discussion schedule was piloted during one interview, after which no changes were deemed necessary. A trained and experienced interpreter was contracted to participate in interviews where the participant did not have sufficient English ability and/or preferred the presence of an interpreter. The interpreter signed a confidentiality agreement prior to data collection and assisted with 10 of the 15 interviews. The interpreter also acted as a cultural broker to the author, for instance, by explaining the meaning behind certain beliefs or traditions mentioned by the participants (Jezewski & Sotnik 2001). Member-checking and reflexivity were employed to improve the credibility and confirmability of research findings throughout data collection.

A voice-recorder was used to record each interview. Upon the completion of data collection, all interviews were transcribed, and translated from isiXhosa to English where necessary, by the same isiXhosa-speaking interpreter used to conduct the interviews. One randomly chosen interpreted interview recording was reviewed against the English transcription and translation by a second isiXhosa first-language-speaking research assistant in order to ascertain the accuracy of the transcript. No major discrepancies were found.

### Data analysis

Data analysis was guided by the phenomenological framework in combination with the framework method of analysis as described by Gale et al. (2013) and thematic analysis as described by Braun and Clarke (2006). The ATLAS.ti (ATLAS.ti 8.3.0 Mac) software programme was used to assist in data management and analysis. Peer debriefing, reflexivity and the creation of an audit trail were methods employed to improve the trustworthiness of the findings.

### Ethical considerations

Ethical clearance for the study was obtained from the Health Research Ethics Committee of Stellenbosch University (reference number: S18/04/068, project ID: 6707) and the Western Cape Health Research Committee. All participants provided informed consent to participate in the study. Participation was voluntary and the identity of the participants was protected throughout the research process.

## Findings and discussion

Seven themes were identified to describe the participants’ overall experience of motherhood. Within these themes, many factors were identified as influencing the participants’ experiences, both positively and negatively.

The seven themes that describe the participants’ overall experiences are as follows:

- Having a preterm infant was ‘difficult’ for all participants.
- Mothers perceived their infants as medically vulnerable.
- The main influencers of parental perception of childhood vulnerability (PPCV) were:
  ■ PPCV was largely influenced by the small size of the infant.
  ■ PPCV was also heavily influenced by medical interventions.
- The presence or absence of maternal support was a large influence on the mothers’ experiences.
- Traditional beliefs influenced and were influenced by prematurity.
- Cultural traditions and healthcare practices were influenced by prematurity.
- Feeding was an overall difficult experience.
- Information sought by participants was best shared interpersonally.

Each theme is described and discussed below. In the discussion, the participants are also referred to as ‘mothers’. Following the discussion of the themes, a summary of the identified factors that influenced the participants’ experiences of early motherhood is presented in a tabular form following Bronfenbrenner’s bio-ecological systems model. The risk factors to the participants’ well-being are indicated (Box 1), along with the factors that protected or assisted the participants’ well-being (Box 2). The influencing factors (see Box 1 and Box 2) are not discussed below as part of the seven larger themes.

BOX 1 Summary of risk factors to maternal well-being, and thus infant development, represented within Bronfenbrenner’s bio-ecological systems theory.

1. **Microsystem:** Maternal perceptions and experiences
  - Threats to maternal health during pregnancy and the preterm birth process increase maternal stress.
  - Negative experiences in the hospital:
    ■ Machines surrounding the infant in the hospital, perceived as infant illness (unwell infants), contributed to maternal stress.
    ■ Tube feeding was perceived as suggesting illness and described as a negative experience.
  - Concerns regarding caregiving at home:
    ■ Concerns regarding feeding the infant according to the prescribed schedules, specifically during the night.
    ■ Concerns about washing the infant safely.
2. **Mesosystem:** Context of the mother and child
  - Hospital context:
    ■ Not all mothers received the same information against traditional medicinal intake within the infant’s first 6 months of life.
    ■ The hospital stay was a period of high maternal stress.
  - Context (familial and physical) of their home:
    ■ Mothers concerned about lack of warmth in homes.
    ■ Lack of caregiving and emotional support at home appeared to negatively affect maternal stress and caregiving experiences.
3. **Exosystem:** The surrounding community
  - Pressure from family and community members to behave in certain ways, for example, seeking traditional healthcare.
  - Maternal reluctance to leave their infants in the care of others, limited sources of caregiving support and therefore was a stress on maternal coping.
  - Affected participation in traditional practices:
    ■ Not participating in Imbekelo could interfere with community interaction, possibly affecting community support.
    ■ Reluctance to travel with the infant for other sources of support (e.g. to grandparents in another province so that they can care for the infant) may be limiting caregiving support.
4. **Macrosystem:** Economic context and governmental policies
  - Financial difficulty:
    ■ Financial difficulties affected the mother’s or family members’ ability to visit at the hospital. Some mothers had to return to work when their infants were about 1 month corrected age.

*Source:* Bronfenbrenner, U., 1989, ‘Ecological systems theory’, in R. Vasta (ed.), *Annals of child development*, vol. 6, pp. 187–248, Jai Press, Grenwich, CT.

BOX 2 Summary of protective factors to maternal well-being, and thus infant development, represented within Bronfenbrenner’s bio-ecological systems theory.

1. **Microsystem:** The mother herself
  - Religious beliefs and practices provided most mothers with a source of support in coping and rationalising their preterm birth experiences.
  - Witnessing their infants’ progress and development throughout and beyond their hospital stays improved mothers’ spirits.
2. **Mesosystem:** Context of the mother and child
  - During hospital stay:
    ■ KMC and educational groups provided caregiving information and experience, as well as support to the mothers.
    ■ The nursing staff were a large source of emotional-, caregiving- and educational support for the mothers, often easing maternal concerns.
    ■ Other mothers in the same hospital wards and groups were found to be a large source of emotional and informational support.
  - Home:
    ■ Support with caring for the infant and household chores, largely provided by close family members such as the infant’s maternal grandmother.
    ■ Emotional support from family, friends and community members proved to have significant influence on maternal coping in general.
    ■ Financial support from family members and partners assisted in easing some caregiving stresses.
    ■ Informational support from family, friends and community members assisted in easing caregiving concerns.
3. **Exosystem:** The surrounding community
  - Community members assisting with caregiving and caregiving advice.
4. **Macrosystem:** Economic context and governmental policies
  - Receiving child grants: Although only discussed by three participants, the grant was sought to provide financial assistance.
  - Maternity leave: All working mothers in the study received maternity leave that allowed them to be with their infants for the first 4 months after birth. This allowed opportunity for all of the above-mentioned protective factors to impact maternal well-being, such as learning about childcare and receiving support from hospital staff and other mothers.

*Source*: Bronfenbrenner, U., 1989, ‘Ecological systems theory’, in R. Vasta (ed.), *Annals of child development*, vol. 6, pp. 187–248, Jai Press, Grenwich, CT.

KMC, kangaroo mother care.

### Theme 1: Having a preterm infant was ‘difficult’ for all participants

Each participant described the experience of having and caring for a preterm infant as ‘difficult’, often using the words ‘scared’, ‘overwhelmed’ and ‘stressful’. Many participants experienced shock and disbelief over the sight of their small infants and had concerns over their infants’ health. A review of related studies revealed similar descriptions of difficult experiences and feelings of disbelief, suggesting that these are universally shared experiences of mothers of preterm infants (Russell et al. 2014; Steyn et al. 2017). These findings emphasise the psychological impact of preterm birth on the mother, highlighting the need for hospital staff to support such new mothers during this time.

### Theme 2: Mothers perceived their infants as medically vulnerable

Most of the participants made comments suggesting that they perceived their infants to be highly vulnerable and were concerned about their well-being. Parental perception of child (infant) vulnerability reflects a parent’s beliefs or attitude regarding their child’s susceptibility to illness or harm (Green & Solnit 1964). High PPCV, believing the child to be more susceptible than most children, is not uncommon with parents of preterm infants (Gordo et al. 2018). High PPCV has also been correlated with high levels of maternal anxiety at discharge and is known to be influenced by the infant’s appearance and changes in parenting roles, often affecting the interaction between parents and infants. Studies have linked high PPCV to worse developmental outcomes within the first year of a preterm infant’s life (Allen et al. 2004). In the current study, the participants’ PPCV was largely influenced by the small size of the infant and by the medical context of the hospital.

#### Subtheme 2.1: Parental perception of childhood vulnerability was largely influenced by the small size of the infant

All mothers remarked feelings of shock or disbelief at their infants’ small size at birth, which appeared to be a cause of stress amongst the mothers. Participant 9 explained, ‘It’s not really nice to have a small baby, you always think about what could go wrong …’. Participants appeared to relate the small size of the infant to caregiving difficulty within different areas (such as washing, feeding or general difficulties in caring for the infant). The small size of the infant and the subsequent high PPCV also appeared to negatively affect other persons in the mother’s life to optimally assist with caring for the infant. Furthermore, high PPCV was found to affect the mother’s routines as one mother was afraid of leaving the house with her infant and another did not want to leave her infant in the care of others, echoing findings of similar studies (Allen et al. 2004).

#### Subtheme 2.2: Parental perception of childhood vulnerability was also heavily influenced by medical interventions

Mothers expressed concerns about their infants’ medical stability both at the hospital and at home. The participants described generally stressful hospital experiences, explaining that seeing their babies in incubators surrounded by loud monitors and tubes caused them to feel stressed, associating the medical interventions with their infant’s medical vulnerability. Participant 16 stated that ‘it was a lot of stress when I saw her in the incubators, but I really didn’t think she was going to make it …’, whilst Participant 5 remarked that she was ‘traumatised’ from her experience in the hospital. These results appear to be widely shared by mothers of preterm infants (Fróes et al. 2020; Steyn et al. 2017). Furthermore, upon discharge many mothers expressed concerns about their infant’s medical vulnerability, which appeared to be negatively influenced by their living conditions. Participant 8 illustrated this when she said:

‘I was excited but afraid [*to go home*], because of, they said here that when you take your baby who is a premature home, it’s easy for her when she to get infected with illnesses out there and everything, infection and everything, I was so scared!’

Furthermore, four of the 15 participants admitted anxieties about the lack of warmth in their homes and the chances of their infants falling ill. A recent study by Boutayeb et al. (2019) found a high infant mortality rate for infants living in low SES circumstances compared to those in higher SES with improved healthcare access, emphasising the influence of social disparities on health. Furthermore, many mothers expressed concerns about keeping to their infants’ feeding schedule, as well as waking up to regularly check their infant’s breathing status. Maternal concerns about feeding their preterm infant at home are found in similar studies (Da Silva et al. 2020).

The voiced maternal concerns suggest the appearance of PPCV, emphasising and confirming the universality of such experiences. With PPCV’s correlation to maternal stress, anxiety and the associated risk of poorer developmental outcomes, it remains imperative that healthcare professionals working with mothers of preterm infants are educated on effective interventions.

### Theme 3: The presence or absence of maternal support was a large influence on the mothers’ experiences

Support from others (with caregiving, information and emotional support) played a significant role in the mothers’ positive experiences. Literature confirms that this is often the case (Leahy-Warren et al. 2020). Those participants who received greater support seemed to have undergone a shift from an initial negative experience to a more positive overall experience. Sources of support in the hospital context included staff (nurses and SLPs) and other mothers of preterm infants experiencing a shared phenomenon. Many participants mentioned kangaroo mother care (KMC) groups and educational groups (run by nurses and allied health professionals) to be positive influences in their experiences. In addition to personal support, religion was found to play a key role in the participants’ meaning-making of the premature birth, and in proving emotional support. Major positive elements of these mothers’ hospital stays were access to religious activities and facilities. Religious aspects of maternal reasoning were also found in a study by Arzani et al. (2015) who explored maternal coping strategies in handling preterm infants. This finding reiterates the importance of recognising an individual’s beliefs and values and the potential influence thereof on their actions and perceptions.

Although all the participants acknowledged numerous sources of support whilst the baby was still in hospital, it was the support in the home environment that had the largest influence on the change in the mothers’ overall experiences. Such valued support often came from the infant’s grandmother in the form of physical caregiving, advice and assistance with household chores. Theme 3 highlights the positive impact of support from healthcare professionals and support facilities (e.g. place of prayer) as well as social networks on maternal experiences.

### Theme 4: Traditional beliefs influenced and were influenced by prematurity

Beliefs about the cause of preterm birth often included both medically influenced understandings and traditional and/or religious beliefs. All but one participant provided a medical reason for the preterm birth of their infants as given to them by the medical doctor at the hospital (e.g. preeclampsia in eight cases), as well as more personal beliefs. Participants also appeared to combine both religious and traditional influences to form a supernatural understanding of their experiences. Participant 6 voiced her religious and/or traditional beliefs as follows: ‘I believe it’s someone bewitched me, I ask God why. If someone, maybe it’s the ancestors they punish me, maybe it’s the god, he is punishing me’. Additionally, two participants talked about their belief that unfaithful partners lead to their children’s preterm births. These findings illustrate the strong influence of both religion and culture or traditional views on personal meaning-making, and are reiterated in similar studies involving participants with different cultures (Elter et al. 2016; Ha et al. 2014; Okafor 2000). The diversity in the findings also highlights the uniqueness of each individual’s perspective. Healthcare professionals, such as doctors, nurses and SLPs, should therefore be sensitive to individuals’ unique perceptions and should strive to provide culturally sensitive and individualised care and interventions.

### Theme 5: Cultural traditions and healthcare practices were influenced by prematurity

The findings suggest that prematurity had an effect on both traditional practices and the practice of traditional healthcare. Two traditions were mentioned during the interviews, namely ‘Imbeleko’ and grandparents’ assistance in raising children. Imbeleko was understood to be a traditional ceremony in which the umbilical cord is buried in a place of significance with the purpose of honouring ancestors for the safe arrival of the infant, as well as introducing the new infant to the clan or community. It is only after the ceremony has taken place that the mother and infant may be visited by their family and community. Because of the participants’ lengthy hospital stay after the preterm birth, the umbilical cord was often discarded by the nursing staff as medical waste. Therefore, many of the participants were not able to perform this traditional ceremony. Participant 7 said that ‘I didn’t have a choice’, suggesting she felt deprived of the opportunity. This highlights the importance of sensitivity by healthcare professionals towards cultural practices, and the consequences of the lack thereof. Furthermore, it was also found that many maternal grandparents assisted in raising the children as part of a community tradition. Most mothers who reported that they would take part in this tradition under ‘normal circumstances’ (i.e. after full-term pregnancy) expressed reluctance to do so in the case of their preterm infant, stating that they would either not send their children to their grandparents at all or would let them go at an older age. The reasons behind these decisions involved preterm infants’ specified caregiving needs for which the mothers had received education during their stay in the hospital with the infant, and high levels of PPCV. Other family members and community members assisting with infant caregiving are also mentioned in other studies involving African cultures (Adama, Sundin & Bayes 2020; Pharr et al. 2014). These findings suggest that healthcare professionals should ensure that caregiver education is accessible to all primary caregivers of infants at the time of discharge and thereafter, and not only the mother.

Furthermore, mothers also showed apprehension in the use of traditional medicines because of their infants’ preterm status. Literature available on the subject of traditional medicine use with infants suggests that there is a common use of traditional medicines in Africa (Kaburi et al. 2015; Nwaiwu & Oyelade 2016). Towns et al. (2014) found that mothers in Western Africa provided their preterm infants with a specific traditional medicine with the aim of enhancing growth. This was not the case with the current study. Fourteen of the 15 participants discussed having previously participated in traditional health practices with other children born at full term in their families. Those practices include ingesting traditional medicines as well as placing a pouch, containing a combination of different herbs, around the infant’s neck or stomach in order to ward off unwanted spirits. The mothers who believed in the protective properties of the herb pouch continued to make use of it with their preterm infants; however, this was not the case with ingesting traditional medicine. According to the participants, most full-term infants in the isiXhosa culture receive a traditional ‘bottle’ of medicine within a few weeks or a month after birth as a form of illness prevention, irrespective of signs or absence of illness. This mixture is reportedly used to prevent and treat stomach upsets (including gas, reflux, irregular stools and general infant unrest). The 14 participants shared different views on the use of the traditional medicines with their preterm infants. Four of the 14 participants gave their preterm infants the traditional medicine to drink with the advice of other family members, traditional healers and their church elders. One participant avoided the ingestible medicine because of her infant’s preterm status and instead used only topical traditional remedies (a mixture of oils and herbs given to her by a traditional healer to ward off unwanted spirits, rubbed onto the infant’s skin), whilst the other nine participants were cautious to use any traditional medicines with their preterm infants and had not used them by the time of data collection. These nine mothers reported being told during their hospital stays that the infants were not to receive medicine (traditional or western) unless directed so by a medical doctor, until the age of 6 months of chronological age. Six of these nine participants reported that, as advised by health professionals, they will only introduce the traditional medicines after the age of 6 months or older, whilst three participants remained undecided on the use of traditional medicines with their preterm-born infants, even though they had used these medicines previously with their full-term babies or family members. It appeared that the preterm status of their infant, as well as the advice from medical professionals, had affected their use of these traditional medicines, as Participant 12 stated: ‘if she was normal I would go to traditional bottles’. The four mothers who used the traditional medicine whilst their infants were still under the age of 6 months did not recall being cautioned against the use of medicines during their hospital stay. These findings illustrate the impact of information provided by medical staff in a hospital setting. Furthermore, this information demonstrates that even those who make use of traditional forms of healthcare also place great importance on advice provided by medical professionals. In the migration towards a culturally competent healthcare system, healthcare professionals should make every effort to respectfully find out about their patients’ traditional healthcare practices and beliefs.

### Theme 6: Feeding was an overall difficult experience

Tube feeding was a largely negative experience, whilst mothers revealed mixed experiences with cup feeding. Of the 15 participants, eight discussed tube feeding. Seven of these eight participants described tube feeding as a negative experience, largely because of the perception that a tube-fed infant is an ‘ill’ infant. It was difficult for the participants to see their infants struggle with the discomfort of the tube. Participant 1 stated that:

‘[*H*]e was really struggling on the tube because it was on his cheek and he would pull it out and they would have to put it back again and it really hurt him.’

Two other participants described their concerns about the tube feeding affecting future feeding abilities, as Participant 5 illustrated:

‘[*T*]he cup was fine, I think the tube was something else, it was overwhelming and I thought she won’t be able to breastfeed and then obviously it would take a while for her to feed, like um with solid food ….’

When the infants were transferred for cup feeding, some mothers found it difficult as they were concerned about their infants ingesting enough milk, whilst other mothers perceived cup feeding as a sign of positive progression. Participant 6 expressed, ‘I felt happy because he was eating, and he is eating the healthy milk’ and Participant 14 shared that ‘it was lovely to see him swallowing on his own’. The participants scarcely mentioned bottle or breast feeding, suggesting that their feeding experiences were generally overpowered by struggling through tube and cup feeding. Stressful feeding experiences and apprehensions with feeding preterm infants appear to be a universally shared parental experience (Lutz 2012; Stevens, Gazza & Pickler 2014). There is an important opportunity within the hospital setting to reduce maternal anxieties regarding tube feeding through education, a vital part of the role of an SLP.

### Theme 7: Information sought by participants was best shared interpersonally

The participants were asked about the sources of the information that they received regarding prematurity, infant caregiving and speech and language development, both during their hospital stay and afterwards at home. All resources reported by the mothers involved persons, as opposed to other sources, such as pamphlets, posters or books. Sources of helpful information for the mothers included the nursing staff, the SLPs (regarding feeding), staff running the KMC groups in the hospital, as well as the other mothers in the wards. Only one mother reported that she had used a book, as well as the Internet, to learn more about prematurity and her infant. Studies by Criss et al. (2015) and Guerra-Reyes et al. (2016) explored health-seeking patterns of mothers in low-income American settings and found that whilst mothers trust and value interpersonal advice from prominent women in their lives (their mothers and community members), they also used their mobile phones. This may be influenced by the differences in technological availability between the two settings.

## Summary

The findings indicate that there are many risk and protective factors affecting a mother’s experience of having, caring for and feeding a preterm infant. Whilst the majority of these experiences appear to be universally shared, others are more context-specific. Important context-specific findings involve cultural beliefs and traditions surrounding preterm birth. Non-standardised approaches to maternal education around the use of traditional medicines with preterm birth illustrate a gap in the understanding thereof, as only some participants were cautioned against traditional medicines where it was not discussed with others. Furthermore, misunderstandings of the risks of tube feeding suggest poor maternal education in the neonatal wards, and point to a vital opportunity for SLPs to intervene and assist in easing maternal stress. Reduced maternal stress may lead to lower PPCV, a known risk for poor infant developmental outcomes.

## Clinical and research implications

It is important for healthcare practitioners working with mothers of preterm infants to understand the specific influences on these caregivers’ lives in order to provide appropriate and respectful care and education. Healthcare practitioners should also know how to sensitively and non-judgementally enquire about mothers’ and other caregivers’ beliefs and practices. Continued in-service training of all hospital staff, but particularly neonatal nurses, doctors and allied health professionals, is therefore of great importance. Additionally, questions pertaining to patients’ beliefs and traditional practices (including space for special requests, such as not to discard the umbilical cord) could be included as part of the admission documentation. This would allow health workers to respect meaningful traditions (for instance to guard against discarding of infants’ umbilical cords), and to guide caregivers in the safe practice of traditional or alternative healthcare.

None of the mothers reported concerns over the risk of developmental delay associated with prematurity, although it is common in children born prematurely. Unawareness of such risk may result in late recognition of developmental difficulties. This was identified as a potential knowledge gap that could be addressed by the SLP, in a manner that would not unnecessarily increase the mother’s anxiety. Furthermore, it is evident that mothers seek and appreciate information during their lengthy hospital stays. Speech-language pathologists thus have an opportunity to provide education about developmental stimulation in order to mitigate possible speech and language delays. Additionally, because tube feeding appeared to be a large stressor for mothers of preterm infants, the SLP has an opportunity to educate the mother and other neonatal intensive care unit (NICU) staff members about tube feeding, address any related concerns and involve the mother with the feeding process where appropriate (something that is strongly encouraged in NICUs worldwide [Craig et al. 2015]). This may assist in reducing the stress and negative perceptions around tube feeding.

Healthcare providers are often unaware of their patients’ cultural beliefs and practices and how these might be dismissed in the routine course of medical intervention. The findings of the current study as well as similar future research involving larger and more diverse patient groups can assist in creating sensitivity regarding the issues that healthcare professionals should consider in order to improve the cultural competence of their services as well as the larger healthcare system, as emphasised in recent literature (Govender et al. 2017; Martin & DiMatteo 2013). Such information will be beneficial to both acute neonatal care settings and broader ECI programmes.

## Limitations

Because of the relatively small group of participants, the study results cannot be generalised to all isi-Xhosa-speaking mothers of preterm infants residing in urban areas and within low socio-economic conditions. However, because of the growing realisation of the importance of research on mother and infant health with consideration of different cultural and social settings, the findings of the study are felt to be important for healthcare professionals working in the South African context, especially for neonatal nurses, doctors and SLPs in the Western Cape province.

## Conclusion

The findings of the current study provide valuable insights into the (not uncommon) experience of mothering a preterm infant in his or her first stage of life within a unique setting in South Africa. The findings highlight the importance of support for maternal coping, the need for maternal education regarding prematurity and early childhood development, as well as the interaction between preterm birth and cultural beliefs and traditions. The findings have implications for healthcare professions (continued) education, maternal support infrastructures, healthcare provided to preterm infants and their mothers in the NICU, as well as caregiver education.
